# Castration of an Elephant

**Published:** 1900-08

**Authors:** Walter Shaw, W. G. King Dodds

**Affiliations:** Dayton, Ohio; Cincinnati, Ohio


### REPORTS OF CASES.

### CASTRATION OF AN ELEPHANT.

By Dr. Walter Shaw,
DAYTON, OHIO,
AND
Dr. W. G. King Dodds,
CINCINNATI, OHIO.

The patient was the property of "The Wallace Circus Co." For many months he was so vicious as to imperil the lives of his attendants. This elephant ("Pilot," by name) was thirty-five years old, an age at which refractoriness should yield to obedience and the animal become docile. Pilot weighed nine thousand pounds and was ten feet tall. His unruly disposition made it dangerous to exhibit him, and the devising of some means to subdue him was imperative. After due deliberation his proprietor decided to have him castrated.

In February, 1899, Mr. Wallace advised us of his decision, and we visited the winter quarters of the circus on his beautiful farm by the classic banks of the Wabash, Peru, Ind. Having surveyed the situation we gave instructions for making excavation, platform, operating-table, and all other arrangements necessary for the completion of the operation, including instruments.

The manner of confining the elephant and the mode of operation are as follows: Having secured two railroad rails on ties placed in the excavation, four large iron bands were fastened around each rail and tie beneath, for the purpose of attaching cable-chains, block and tackle; the space between the rails was filled with cement and this was overlaid with heavy oak planks. His caretaker placed a large cable-chain around the elephant's girth, in which was a strong ring just below his chest. To this ring another cable-chain was fastened and passed through a ring safely attached to his tusk, then about the anterior of trunk, through another ring on opposite tusk back to ring immediately under the body. In addition to this (as a guarantee of safety), a strong rope was passed several times around his trunk and tusks. In this condition he was conducted on the platform which he held in place by means of his own weight. To each foot a large cable-chain was secured slightly slack, this was accomplished to warrant safety in the event that the block and tackle would break. His front feet were then drawn forward and outward, and his hind feet were drawn backward and outward by means of block and tackle. At this juncture the operating-table, four feet wide and sixteen feet long, was arranged beneath his body; then his feet were extended until his body came in contact with the table slightly canted to right side. A rope was attached to each tusk and fastened at right angles.

As he was put under the influence of an anæsthetic the utmost caution was exercised. When insensible, an incision was made in his left side through the dermis just posterior to last rib, beginning at the end of the transverse process of lumbar vertebræ, extending downward about ten inches. The skin was an inch thick, white and almost bloodless; the external three-fourths of an inch was very viscous and difficult to cut. An eight-inch opening was made through the muscles and peritoneum, in which the hand was introduced, bearing forward and finding the glands just posterior to the kidneys. These glands were loosely but securely attached. The emasculator, which was made with a screw to remove the glands, broke before either gland was displaced, because it was too highly tempered, otherwise it would have answered the purpose. This misfortune made it necessary to reach in at arm's length and cut the ligature, the best possible way under the circumstances, and remove the glands by the aid of the knife. The peritoneum, muscles and skin were sutured in the usual manner, and the incision was closed with wound gelatin. The operation was made aseptic so far as possible. The influence of the anæsthetic had waned by the time the operation was completed, and the animal did not seem to be distressed.

He lived fifty-seven hours, during which time his appetite was normal, did not seem to suffer until an hour before his death. The direct cause of his death is not known, as no examination was made. With the right kind of instruments properly constructed, this operation, which required an hour and a-half, could have been accomplished in thirty minutes. Under favorable conditions, and with suitable instruments, we believe that the operation could be successfully performed, as it would then not be necessary to have the abdominal cavity open more than ten or fifteen minutes.

